# Rear textured p-type high temperature passivating contacts and their implementation in perovskite/silicon tandem cells†

**DOI:** 10.1039/d3ya00048f

**Published:** 2023-09-26

**Authors:** Arnaud Walter, Brett A. Kamino, Soo-Jin Moon, Patrick Wyss, Juan J. Diaz Leon, Christophe Allebé, Antoine Descoeudres, Sylvain Nicolay, Christophe Ballif, Quentin Jeangros, Andrea Ingenito

**Affiliations:** a CSEM SA, Sustainable Energy Jaquet-Droz 1 2002 Neuchâtel Switzerland andrea.ingenito@csem.ch; b Institute of Electrical and Microengineering (IEM), Photovoltaics and Thin-Film Electronics Laboratory (PV-Lab), Ecole Polytechnique Fédérale de Lausanne (EPFL) Rue de la Maladière 71b 2002 Neuchâtel Switzerland

## Abstract

Silicon solar cells based on high temperature passivating contacts are becoming mainstream in the photovoltaic industry. Here, we developed a high-quality boron-doped poly-silicon hole contact. When combined with a co-processed phosphorus-doped poly-silicon electron contact, high-voltage silicon bottom cells could be demonstrated and included in 28.25%-efficient perovskite/Si tandems. The active area was 4 cm^2^ active area and the front electrode was screen-printed.

## Abstract 2

High temperature passivating p-type contacts on textured surfaces with i*V*_OC_ up to 725 mV and contact resistance below 90 mΩ cm^2^. 28.25% tandem PK/c-Si solar cells rear side textured with high temperature passivating contacts.

## Graphical abstract

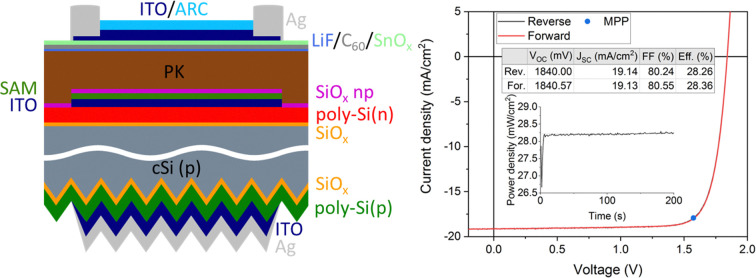

With efficiencies recently overcoming the 30% mark with laboratory-scale solar cells,^1–3^ perovskite/Si (PK/Si) tandem cells are increasingly considered a credible candidate for high-efficiency commercial PV and the technology starts to be part of the development roadmap of most crystalline Si (c-Si) solar cell manufacturers. However, nearly all of the highest efficiency tandem devices reported to date rely on a Si heterojunction (SHJ) bottom cell.^4–6^ At the industrial level, the SHJ technology occupies only a small niche of a market moving from the passivated emitter and rear cell (PERC)^7,8^ concept towards the use of the tunnel oxide-passivating contact (TOPCon) technology, which is based on high temperature passivating contacts (HTPC).^8,9^ In TOPCon devices, the cells and contacts are processed at higher temperature enabling the use of equipment and processes more similar to the PERC technology, along with lower quality wafers. In view of a future industrial deployment, the demonstration of a PK/Si cell built upon more widely used processes or device architectures is hence needed. Such tandems have been previously produced using industrially-relevant bottom cells based on the PERx/TOPCon technology or a modified TOPCon with rear localized contacts.^7,10^ These devices reached efficiencies of 28.7% and 27.6%, respectively, on a 1 cm^2^ active area. However, these designs suffer from limited passivation at the rear of the c-Si cell, and, in the second case, from an increased fabrication complexity. More recently, a tandem cell based on a TOPCon c-Si cell featuring a p-type rear contact deposited on a textured wafer was shown to reach an efficiency of 28.2%, albeit on 0.124 cm^2^ and on an n-type wafer.^11^ Here, we demonstrate a 4 cm^2^ >28%-efficient tandem cell featuring full-area HTPC deposited on both sides of the Si wafer. Noticeably, the rear-side poly-SiC_*x*_(p) hole selective contact is deposited by plasma-enhanced chemical vapor deposition (PECVD) on a KOH-etched textured surface to maximize the infrared light response (light trapping) of the bottom c-Si cell. Reaching sufficient passivation quality and hole transport properties has proven challenging on textured c-Si surfaces, limiting our previous demonstrations of perovskite/Si HTPC tandems to designs featuring a flat poly-SiC_*x*_(p) contact at the rear of the bottom cell.^12^ Indeed, surface passivation is known to be weaker in p-type poly-Si/SiO_*x*_ stack, compared with the n-type poly-Si/SiO_*x*_ stack.^13–22^ This difference has been shown to be even more dramatic on textured surfaces,^21–23^ possibly due to the poorer passivation quality at the 〈111〉 surface-oriented plane (textured) compared to the 〈100〉 (planar).^22^ The 28%-device presented in the current work also benefits from previous work^24^ on low-temperature metallization screen-printing processes, which are used in the c-Si photovoltaic industry.

To assess the potential of the newly developed p-type contact, symmetric, both-sides textured samples were fabricated on p-type c-Si wafers. Textured float zone (FZ) p-type wafers (4′′) were used with a thickness of ∼190 μm and a resistivity of ∼2 Ω cm. After standard wafer cleaning, a ∼1.2 nm-thick SiO_*x*_ layer was grown by plasma enahncec chemical vapor deposition (PECVD). A boron-doped silicon carbide (SiC_*x*_(p)) film with a thickness of 45 nm (on flat) was symmetrically deposited by PECVD. The samples were then annealed in a tube furnace at 850 °C, typically with a dwell time of 15 min. This treatment is required to crystallize the SiC_*x*_ contact and promote the diffusion of dopants from the contact to the Si wafer. After annealing, SiN_*x*_:H was deposited by PECVD, followed by firing at 800 °C in an inline furnace for the hydrogenation of defects. After stripping of the SiN_*x*_:H in HF, ITO was deposited by sputtering through a hard metallic mask to define the contact geometry used for contact resistance (*ρ*_c_) measurements. Fig. 1(a) and (b) reports the implied open circuit voltage (i*V*_OC_) and the specific contact resistance (*ρ*_c_) measured on symmetric textured samples as a function of the CH_4_ flow used to deposit the SiC_*x*_(p) contact by PECVD (normalized to its maximum value, CH_4_/CH_4 Normalized_max_). Excellent i*V*_OC_ of 725 mV and *ρ*_c_ below 90 mΩ cm^2^ were obtained for a CH_4 Normalized_max_ = 0.5 and for an annealing temperature of 850 °C for a dwell time of 15 min. To our knowledge these are among the best values reported so far for ap-type HTPC on a textured Si substrate.^22,23^

**Fig. 1:**
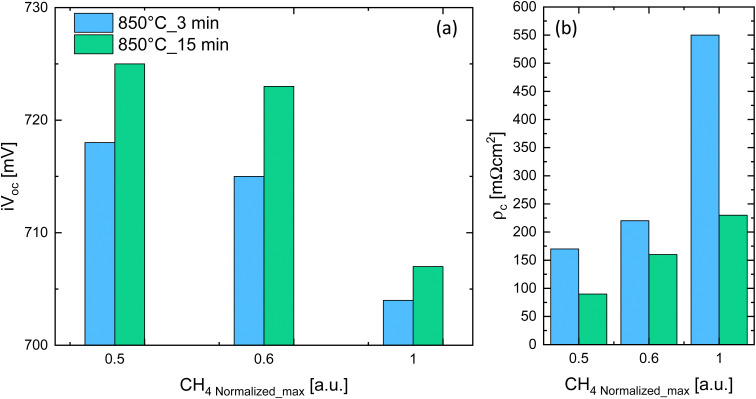
(a) Implied open circuit voltage (i*V*_OC_) and (b) specific contact resistance (*ρ*_c_) as a function of the CH_4_ flow normalized to the maximum value (CH_4_/CH_4 max_), CH_4 Normalized_max_. The SiC_*x*_(p) is deposited here on both sides of textured wafers.

The decrease in i*V*_OC_ and increase in *ρ*_c_ towards higher CH_4_ flows is explained by the higher amount of C that can disrupt the interfacial SiO_*x*_ and lowers B-doping incorporation for films richer in C.^25,26^ We hypothesize that the lower i*V*_OC_ and higher *ρ*_c_ for annealing dwell time of 3 min compared to 15 min (for the same normalized CH_4_ flow) is explained by a shallower B in-diffused region into the c-Si wafer.^12,14,17,27–31^ Rear-side textured HTPC bottom cells were processed on single-side textured FZ p-type wafers (4′′) of the same thickness and base resistivity as the symmetrical samples. On the rear-side, the above-optimized SiC_*x*_(p) was deposited while a SiC_*x*_(n) as reported by Ingenito *et al.*^25^ was used for the front planar side. After stripping in HF of the SiN_*x*_:H used to hydrogenate the contact, i*V*_OC_ values up to 723 mV are obtained on p-type wafers.

Taking advantage of the superior passivating properties of this new p-contact on textured c-Si wafers, PK/Si tandems were fabricated. Fig. 2(a) shows schematically the perovskite/Si HTPC device. Following the stripping of the SiN_*x*_, the ITO/Ag back electrode was created by sputtering. For the monolithic interconnection of the tandem sub-cells, a 10 nm-thin ITO recombination junction was sputtered onto the front SiC_*x*_(n) through a shadow mask. The top cell absorber had a composition of Cs_0.17_FA_0.83_Pb(I_0.83_Br_0.17_)_3_, corresponding to a bandgap of 1.63 eV. Interface recombination plays a significant role in perovskite solar cells, affecting both ideality factor and *V*_OC_.^32^ Similar to Al-Ashouri *et al.*,^33^ we employ a self-assembled monolayer (SAM) made of Me-4PACz as the hole transport layer (HTL), enabling excellent interface passivation and hole selectivity. A wetting layer made of SiOx nanoparticles was added. On the Electron transport layer (ETL) side, a thin layer of LiF is thermally evaporated between the perovskite and the C_60_ ETL. It is to be noted that the perovskite absorber was deposited on the full area of the 4′′ Si wafer, with each 4 cm^2^ tandem being then defined by the deposition of the front ITO electrode through a 7-cell metal mask. A Ag paste was then screen-printed at low temperature to metallize the cell,^24^ before depositing a LiF antireflective coating (ARC). Fig. 2(b) shows a picture of the 7 tandem cells on a 4′′ wafer.

**Fig. 2:**
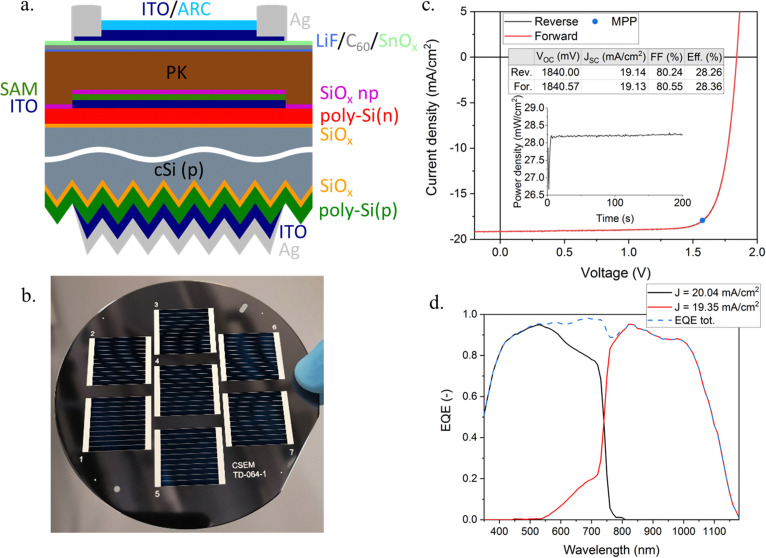
(a) Schematic view of the tandem cell stack. (b) Image of 7 tandem cells on a 4” wafer. The metallization is done by screenprinting. (c) Current–voltage (*JV*) characteristics of the best performing 4 cm^2^ cell. The inset table shows the *JV* parameters of the reverse (*V*_OC_ → *J*_SC_, black) and forward (*J*_SC_ → *V*_OC_, red) scans. The inset graph provides the maximum power point (MPP) tracking over 200 s, showing a steady-state efficiency of 28.25%. (d) External quantum efficiency (EQE) spectra of the top perovskite (black) and bottom Si HTPC (red) sub-cells. The blue dashed line shows the sum of the two.

The current–voltage (*JV*) characteristics of the tandem cells were measured using a calibrated two-lamp solar simulator. The *JV* curve of the best device is displayed in Fig. 2(c). The cell was measured in the reverse (from *V*_OC_ to *J*_SC_) and in the forward (from *J*_SC_ to *V*_OC_) directions, highlighting a negligible hysteresis in these scan rate conditions (0.19 V s^−1^). The reverse *JV* measurement yields a *V*_OC_ of 1840 mV, a *J*_SC_ of 19.14 mA cm^−2^ and a Fill factor (FF) of 80.24%, resulting in a PCE of 28.26%, a value on par with record values reported with 4 cm^2^ perovskite/SHJ cells.^34^ This result is confirmed by a maximum power point tracking (MPP) over 200 s, which converges to a maximum power density of 28.25 mW cm^−2^ (average power over the last 60 s of tracking), as can be seen in the inset of Fig. 2(c). Fig. 2(d) shows the EQE measurement of the two sub-cells. From the integrated current density, one can infer that, with a top cell current of 20.04 mA cm^−2^ and a bottom cell current of 19.35 mA cm^−2^, the tandem device power output is limited by the bottom cell current, which may hence explain the high FF of the device^35^ (>80%). The monochromatic light spot of the EQE measurement is focused between the silver lines of the front metallization, thus excluding shadow losses and hence explaining the discrepancy with the *J*_sc_ from the *JV* curve. Fig. S1 of the ESI† shows the distribution of the *JV* parameters across the 4′′ Si wafer, demonstrating the good uniformity over a large area.

Thanks to the scalability of the different techniques involved – and notably of the metallization, we have successfully upscaled the process for perovskite/silicon tandem devices to a 4′′ pseudo-square with an active area of 57.4 cm^2^. The device exhibited a PCE of 22.51%. The perovskite layer and the front grid metallization were deposited using the same deposition approach as for the small area devices. These results demonstrate the potential for further optimization and scaling of perovskite/silicon tandem devices. This result is summarized in Fig. S2 (ESI†).

To conclude, we report here a perovskite/Si tandem solar cell based on a both-side passivating contact bottom cell architecture with a poly-Si(p) passivating contact deposited on the textured rear-side of the Si cell. This device reaches a power conversion efficiency of 28.25% on an active area of 4 cm^2^, in part thanks to a FF above 80%. These results demonstrate that Si cells based on HTPC, a category of contacts that is gaining traction on the PV market, can be combined with a perovskite top cell to reach efficiencies comparable to these reached with perovskite/SHJ tandems, and are also scalable to larger active area than those typically reported in the literature (1 cm^2^).

## Author contributions

A. W. and A. I.: conceptualization, data curation, formal analysis, investigation, methodology, validation, visualization, writing – original draft; P.W.: investigation and resources; B. A. K., S.-J. M. J. J. D.L., C. A. and A. D.: conceptualization, investigation, methodology, validation; S. N and Q. J.: funding acquisition, project administration and supervision; C. B.: supervision. All: writing – review and editing.

## Conflicts of interest

There are no conflicts to declare.

## Supplementary Material

YA-002-D3YA00048F-s001
