# Supplementary material for: Rear textured p-type high temperature passivating contacts and their implementation in perovskite/silicon tandem cells
Source: Energy Adv. 2023 Sep 26;2(11):1818–22. doi: 10.1039/d3ya00048f (PMC10634458; doi:10.1039/d3ya00048f)
Supplement: YA-002-D3YA00048F-s001 [file YA-002-D3YA00048F-s001.pdf]

Electronic Supplementary Material (ESI) for Energy Advances.
This journal is © The Royal Society of Chemistry 2023

ELECTRONIC SUPPLEMENTARY INFORMATION

Rear Textured p-type High Temperature Passivating Contacts and their Implementation in Perovskite/Silicon Tandem Cells

Arnaud Walter,[a,] Brett A. Kamino,[a] Soo-Jin Moon,[a] Patrick Wyss,[a] Juan J. Diaz Leon,[a] Christophe Allebé,[a] Antoine Descoeudres,[a] Sylvain Nicolay,[a,†] Christophe Ballif,[a,b] Quentin Jeangros,[a] Andrea Ingenito[a]*

AUTHOR ADDRESS

[a] CSEM SA, Sustainable Energy Center, Jaquet-Droz 1, 2002 Neuchâtel, Switzerland

[b] Institute of Electrical and Microengineering (IEM), Photovoltaics and Thin-Film Electronics Laboratory (PV-Lab), Ecole Polytechnique Fédérale de Lausanne (EPFL), Rue de la Maladière 71b, 2002 Neuchâtel, Switzerland

AUTHOR INFORMATION

[†]now at: Institut Interdisciplinaire d'Innovation Technologique (3IT), Sherbrooke University, Sherbrooke, Canada

Corresponding Author

*arnaud.walter@csem.ch

Fabrication details

HTPC bottom cells development: Symmetrical samples were first realized on n- and p-type wafers to assess the potential of the passivating contacts on both wafer polarities and to optimize the tunnel oxide growth. For that purpose, planar float zone (FZ) n-type and textured FZ p-type wafers (4”) were used with a thickness of ~190 µm and a resistivity of ~2 Ω·cm. After standard wafer cleaning, a ~1.2 nm-thick SiO_x layer was grown by UV-O_3 exposure. On n-type wafers, a phosphorus-doped silicon layer with few %at of carbon (SiC_x(n)) with a thickness of 35 nm was symmetrically deposited by plasma enhanced chemical vapor deposition (PECVD). On p-type textured wafers, a boron-doped silicon carbon (SiC_x(p)) film with a thickness of 45 nm (on flat) was symmetrically deposited by PECVD. Both layers were annealed in a tube furnace at 850°C, typically with a dwell time of 15 min. After annealing, SiN_x:H was deposited by PECVD, followed by firing at 800°C in an inline furnace. After stripping of the SiN_x:H in HF, ITO was deposited by sputtering through a hard metallic mask to define the contact geometry used for contact resistance (ρ_c) measurements. For solar cell fabrication, we used FZ n-type and p-type wafers (4”) that are single-side textured with a thickness of ~190 µm and a resistivity of ~2 Ω·cm. After standard wafer cleaning and SiO_x growth, SiC_x(p) was deposited on the rear textured side and SiC_x(n) on the planar front side. Same annealing, firing, and SiN_x processing steps as for the symmetrical test samples were applied.

Perovskite top cell deposition and tandem processing: 2-terminal PK/Si HTPC tandem cells were prepared on rear-side textured bottom cells. A thin, 10 nm, ITO recombination junction was deposited by sputtering through a shadow mask on the front SiC(n). The hole transport layer consisted in a Self-Assembled Monolayer of Me-4PACz deposited by spin-coating from a 1 mM solution in ethanol, followed by an annealing at 100°C for 10 min. Subsequently a solution of SiO_2 nanoparticle dispersed in ethanol was deposited on top of the SAM by spin-coating to improve the wetting of the perovskite ink.[1,2] The perovskite (PK) absorber was deposited via spin-coating following an anti-solvent route similar to that described by B. A. Kamino *et al.*[3]. An electron transport layer (ETL) stack of LiF/C60 was thermally evaporated on top of the perovskite, followed by atomic layer deposition (ALD) of a SnO_x buffer layer (~10 nm). A 65 nm-thick ITO contact was then sputtered through a shadow mask. Finally, a low-temperature screen-printed Ag metallization was applied to finish the front contact and ~100 nm of LiF was thermally deposited as the antireflective coating. All cells were processed on full 4” Si bottom cells. The active area of the tandems is defined by the size of the electrodes.

Device characterization

A Sinton WCT-120 system was used to measure the minority carrier lifetime (τ_{eff}) and to determine the implied open circuit voltage (iV_{OC}) of the bottom cells. Spatial homogeneity of the passivation was observed using photoluminescence imaging (PLI). Transfer length measurements were used for ρ_c measurements.

Illuminated *JV* curves were recorded on a large-area class A+A+A+ solar simulator from WACOM using a two-light source (xenon and halogen). The solar simulator light intensity was verified with an externally calibrated reference cell to match the 1-sun AM1.5G equivalent intensity. The spectral balance was then checked by means of two filtered externally calibrated

cells to ensure proper balance between the two sub-cells of the tandem. All measurements were done using a four-point method. All cells were measured on a metallic vacuum chuck with an active temperature control set to 25°C. Cells areas were masked with laser cut aperture masks whose opened areas were optically measured. All cells were measured from 1.9 V to -0.2 V and from -0.2 V to 1.9 V with a scan rate of ~191 mV/s. Maximum power point tracking was performed using an in-house tracking algorithm which actively modifies the voltage.

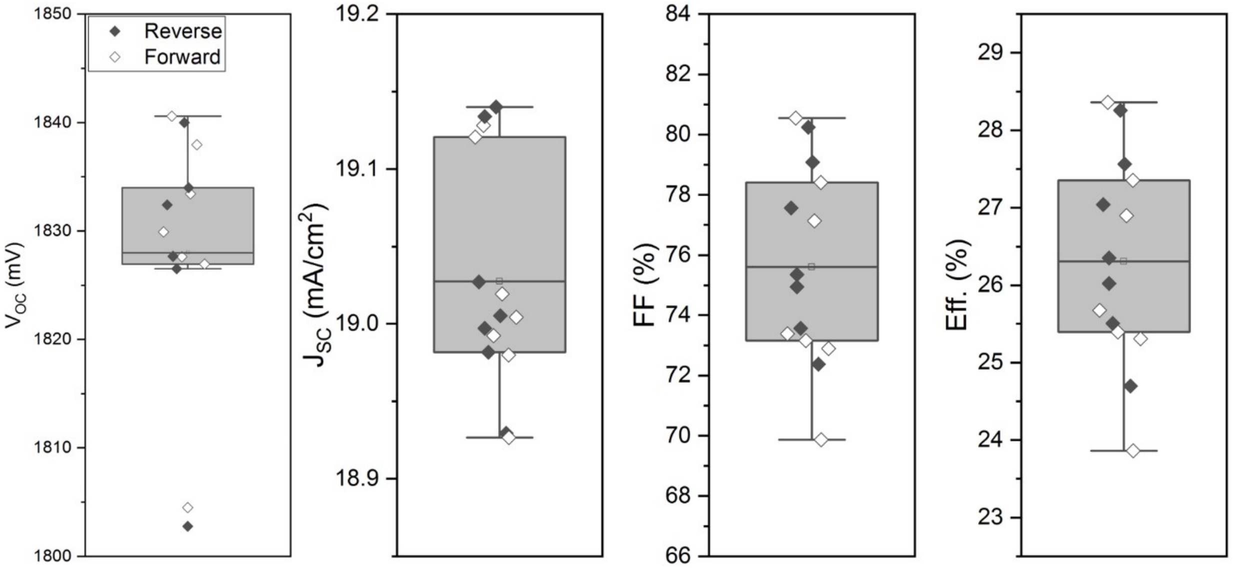

Figure S1. Distribution of *JV* parameters across a 4" wafer (7 cells, each cell is 2x2 cm^2).

External quantum efficiency measurements were made using a custom setup made in house. The calibration of the EQE was made using an externally calibrated cell. EQE measurements for the cells were made at a chopping frequency of approximately 230 Hz. Blue and red light biases were used to saturate the top and bottom cells, respectively.

Large area tandem

A large area tandem was realized using the same type of bottom HTPC cell. For this, the top cell was deposited following the same procedure described above. The larger active area was simply defined by using a large area shadow mask for the deposition of the front ITO contact, followed by the screen printing of a full area metallization. The total active area is 57.4 cm^2.

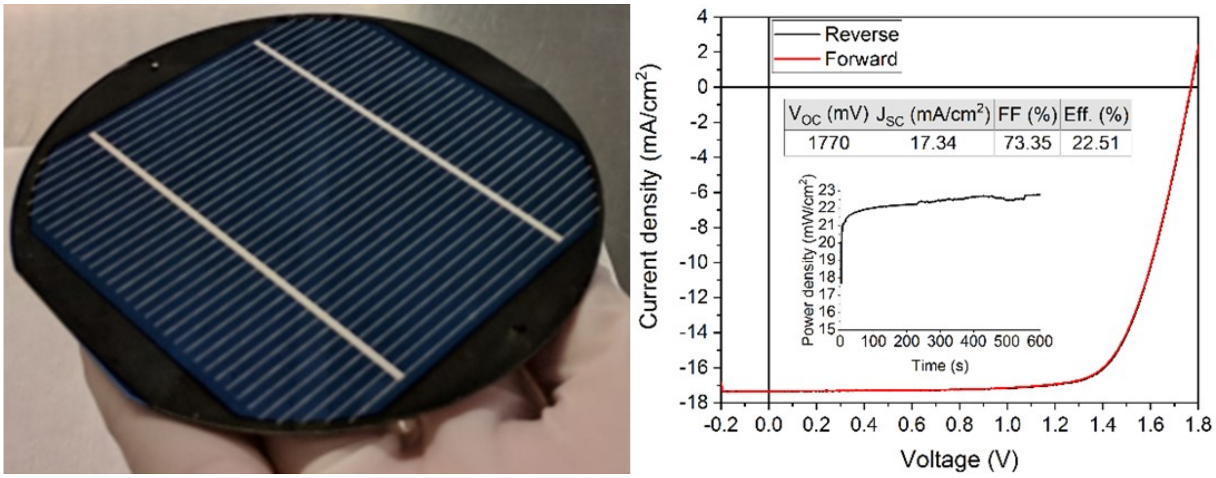

Figure S2. Picture of a large area tandem on a 4” wafer. The active area is 57.4 cm^2 (left). *JV* curve and MPP tracking (inset) of a corresponding tandem device (right).

Table S1: Selection of non-SHJ 2T tandems reported to date

ID	Year	Institute	Bottom cell type	Texture	wafer polarity	Polarity	PK deposition method	Voc (V)	Jsc (mA/cm^2)
1	2015	Stanford/MIT	Al BSF	Flat	n	n-i-p	Spin Coating	1.65	11.5
2	2016	EPFL/CSEM	Al BSF	Flat	n	n-i-p	Spin Coating	1.64	15.3
3	2017	ANU	Homojunction	Flat	n	n-i-p	Spin Coating	1.75	17.6
4	2018	UNSW	Homojunction	Flat	n	n-i-p	Spin Coating	1.68	16.1
5	2018	UNSW	Homojunction	Flat	n	n-i-p	Spin Coating	1.66	15.6
6	2018	UNSW	Homojunction	Flat	n	n-i-p	Spin Coating	1.74	16.2
7	2018	ANU	POLO	Flat	n	p-i-n	Spin Coating	1.76	17.8
8	2019	UNIST/KIST	Al BSF	Flat	p	p-i-n	Spin Coating	1.65	16.1
9	2019	UNIST	Al BSF	Textured	p	n-i-p	Spin Coating	1.59	15.48
10	2019	UNSW	Al BSF	Flat	n	n-i-p	Spin Coating	1.73	16.5
11	2019	EPFL/CSEM	HTPC/Topcon	Textured	p	p-i-n	Spin Coating	1.74	19.5
12	2022	HZB/HQC	PERC	Flat	p	p-i-n	Spin Coating	1.907	19.29
13	2022	ISFH/HZB	PERC/Topcon	Flat	p	p-i-n	Spin Coating	1.8	17.07
14	2022	ANU	PERC/Topcon	Flat	n	p-i-n	Spin Coating	1.794	19.68
15	2022	Korea University	HTPC/Topcon	Flat	n	n-i-p	Spin Coating	1.84	14.4
16	2022	CAS	HTPC/Topcon	Textured	n	p-i-n	Spin Coating	1.744	19.38
17	2022	Ningbo	HTPC/Topcon	Textured	n	p-i-n	Spin Coating	1.797	19.39
18	2023	Ningbo	HTPC/Topcon	Flat	n	p-i-n	Spin Coating	1.78	18.8
**19**	**2023**	**CSEM**	**HTPC/Topcon**	**Flat**	**p**	**p-i-n**	**Spin Coating**	**1.84**	**19.13**

1 D. Turkay, K. Artuk, X.-Y. Chin, D. A. Jacobs, S.-J. Moon, A. Walter, M. Mensi, G. Andreatta, N. Blondiaux, H. Lai, F. Fu, M. Boccard, Q. Jeangros, C. M. Wolff and C. Ballif, High-efficiency (>30%) monolithic perovskite-Si tandem solar cells with flat front-side wafers, *Nat. Energy*.

2 M. Schultes, N. Giesbrecht, J. Küffner, E. Ahlswede, P. Docampo, T. Bein and M. Powalla, Universal Nanoparticle Wetting Agent for Upscaling Perovskite Solar Cells, *ACS Appl. Mater. Interfaces*, 2019, **11**, 12948–12957.

3 B. A. Kamino, B. Paviet-Salomon, S. Moon, N. Badel, J. Levrat, G. Christmann, A. Walter, A. Faes, L. Ding, J. J. Diaz Leon, A. Paracchino, M. Despeisse, C. Ballif and S. Nicolay, Low-Temperature Screen-Printed Metallization for the Scale-Up of Two-Terminal Perovskite–Silicon Tandems, *ACS Appl. Energy Mater.*, 2019, **2**, 3815–3821.

4 J. P. J. P. Mailoa, C. D. C. D. Bailie, E. C. E. C. Johlin, E. T. E. T. Hoke, A. J. A. J. Akey, W. H. W. H. Nguyen, M. D. M. D. McGehee and T. Buonassisi, A 2-terminal perovskite/silicon multijunction solar cell enabled by a silicon tunnel junction, *Appl. Phys. Lett.*, 2015, **106**, 121105.

5 J. Werner, A. Walter, E. Rucavado, S.-J. Moon, D. Sacchetto, M. Rienaecker, R. Peibst, R. Brendel, X. Niquille, S. De Wolf, P. Löper, M. Morales-Masis, S. Nicolay, B. Niesen and C. Ballif, Zinc tin oxide as high-temperature stable recombination layer for mesoscopic perovskite/silicon monolithic tandem solar cells, *Appl. Phys. Lett.*, 2016, **109**, 233902.

6 Y. Wu, D. Yan, J. Peng, T. Duong, Y. Wan, S. P. Phang, H. Shen, N. Wu, C. Barugkin, X. Fu, S. Surve, D. Grant, D. Walter, T. P. White, K. R. Catchpole and K. J. Weber, Monolithic perovskite/silicon-homojunction tandem solar cell with over 22% efficiency, *Energy Environ. Sci.*, 2017, **10**, 2472–2479.

7 J. Zheng, J. Lau, H. Mehrvarz, F. Ma, Y. Jiang, X. Deng, A. Soeriyadi, J. Kim, M. Zhang, L. Hu, X. Cui, D. S. Lee, J. Bing, Y. Cho, C. Chen, M. Green, S. Huang and A. W. Y. Ho-Baillie, Large area efficient interface layer free monolithic perovskite/ homo-junction-silicon tandem solar cell with over 20% efficiency, *Energy Environ. Sci.*, 2018, 0–41.

8 J. Zheng, H. Mehrvarz, F.-J. Ma, C.-F. J. Lau, M. Green, S. Huang and A. W. Y. Ho-Baillie, 21.8% Efficient Monolithic Perovskite/Homo-Junction-Silicon Tandem Solar Cell on 16 cm2, *ACS Energy Lett.*, 2018, **3**, acsenergylett.8b01382.

9 H. Shen, S. T. Omelchenko, D. A. Jacobs, S. Yalamanchili, Y. Wan, D. Yan, P. Phang, T. Duong, Y. Wu, Y. Yin, C. Samundsett, J. Peng, N. Wu, T. P. White, G. G. Andersson, N. S. Lewis and K. R. Catchpole, In situ recombination junction between p-Si and TiO2 enables high-efficiency monolithic perovskite/Si tandem cells, *Sci. Adv.*, 2018, **4**, eaau9711.

10 C. U. Kim, J. C. Yu, E. D. Jung, I. Y. Choi, W. Park, H. Lee, I. Kim, D.-K. Lee, K. K. Hong, M. H. Song and K. J. Choi, Optimization of device design for low cost and high efficiency planar monolithic perovskite/silicon tandem solar cells, *Nano Energy*, 2019, **60**, 213–221.

11 I. Y. Choi, C. U. Kim, W. Park, H. Lee, M. H. Song, K. K. Hong, S. Il Seok and K. J. Choi, Two-terminal mechanical perovskite/silicon tandem solar cells with transparent conductive adhesives, *Nano Energy*, 2019, **65**, 104044.

12 J. Zheng, H. Mehrvarz, C. Liao, J. Bing, X. Cui, Y. Li, V. R. Gonçales, C. F. J. Lau, D. S. Lee, Y. Li, M. Zhang, J. Kim, Y. Cho, L. G. Caro, S. Tang, C. Chen, S. Huang and A. W. Y. Ho-Baillie, Large-Area 23%-Efficient Monolithic Perovskite/Homojunction-Silicon Tandem Solar Cell with Enhanced UV Stability Using Down-Shifting Material, *ACS Energy Lett.*, 2019, 2623–2631.

13 G. Nogay, F. Sahli, J. Werner, R. Monnard, M. Boccard, M. Despeisse, F.-J. F.-J. F.-J. Haug, Q. Jeangros, A. Ingenito and C. Ballif, 25.1%-Efficient Monolithic Perovskite/Silicon Tandem Solar Cell Based on a p-type Monocrystalline Textured Silicon Wafer and High-Temperature Passivating Contacts, *ACS Energy Lett.*, 2019, **4**, 844–845.

14 K. Sveinbjörnsson, B. Li, S. Mariotti, E. Jarzembowski, L. Kegelmann, A. Wirtz, F. Frühauf, A. Weihrauch, R. Niemann, L. Korte, F. Fertig, J. W. Müller and S. Albrecht, Monolithic Perovskite/Silicon Tandem Solar Cell with 28.7% Efficiency Using Industrial Silicon Bottom Cells, *ACS Energy Lett.*, 2022, 2654–2656.

15 S. Mariotti, K. Jäger, M. Diederich, M. Sophie Härtel, B. Li, K. Sveinbjörnsson, S. Kajari-Schröder, R. Peibst, S. Albrecht, L. Korte and T. Wietler, Monolithic Perovskite/Silicon Tandem Solar Cells fabricated using industrial p-type POLO/PERC Silicon Bottom Cell Technology, *Sol. RRL*, 2022, **2101066**, 1–9.

16 Y. Wu, P. Zheng, J. Peng, M. Xu, Y. Chen, S. Surve, T. Lu, A. D. Bui, N. Li, W. Liang, L. Duan, B. Li, H. Shen, T. Duong, J. Yang, X. Zhang, Y. Liu, H. Jin, Q. Chen, T. White, K. Catchpole, H. Zhou and K. Weber, 27.6% Perovskite/c-Si Tandem Solar Cells Using Industrial Fabricated TOPCon Device, *Adv. Energy Mater.*, 2022, **2200821**, 2200821.

17 J. Y. Hyun, K. M. Yeom, S. W. Lee, S. Bae, D. Choi, H. Song, D. Kang, J. K. Hwang, W. Lee, S. Lee, Y. Kang, H. S. Lee, J. H. Noh and D. Kim, Perovskite/Silicon Tandem Solar Cells with a Vocof 1784 mV Based on an Industrially Feasible 25 cm2 TOPCon Silicon Cell, *ACS Appl. Energy Mater.*, , DOI:10.1021/acsaem.1c02796.

18 Z. Ying, Z. Yang, J. Zheng, H. Wei, L. Chen, C. Xiao, J. Sun, C. Shou, G. Qin, J. Sheng, Y. Zeng, B. Yan, X. Yang and J. Ye, Monolithic perovskite/black-silicon tandems based on tunnel oxide passivated contacts, *Joule*, 2022, 1–18.

19 J. Zheng, H. Wei, Z. Ying, X. Yang, J. Sheng, Z. Yang, Y. Zeng and J. Ye, Balancing Charge-Carrier Transport and Recombination for Perovskite/TOPCon Tandem Solar Cells with Double-Textured Structures, *Adv. Energy Mater.*, 2022, **2203006**, 2203006.

20 X. Wang, Z. Ying, J. Zheng, X. Li, Z. Zhang, C. Xiao, Y. Chen, M. Wu, Z. Yang, J. Sun, J.-R. Xu, J. Sheng, Y. Zeng, X. Yang, G. Xing and J. Ye, Long-chain anionic surfactants enabling stable perovskite/silicon tandems with greatly suppressed stress corrosion, *Nat. Commun.*, 2023, **14**, 2166.
